# Clinical, Immunological, and Molecular Profile of Chronic Granulomatous Disease: A Multi-Centric Study of 236 Patients From India

**DOI:** 10.3389/fimmu.2021.625320

**Published:** 2021-02-25

**Authors:** Amit Rawat, Pandiarajan Vignesh, Murugan Sudhakar, Madhubala Sharma, Deepti Suri, Ankur Jindal, Anju Gupta, Jitendra Kumar Shandilya, Sathish Kumar Loganathan, Gurjit Kaur, Sanchi Chawla, Pratap Kumar Patra, Alka Khadwal, Biman Saikia, Ranjana Walker Minz, Vaishali Aggarwal, Prasad Taur, Ambreen Pandrowala, Vijaya Gowri, Mukesh Desai, Manasi Kulkarni, Gauri Hule, Umair Bargir, Priyanka Kambli, Manisha Madkaikar, Sagar Bhattad, Chetan Ginigeri, Harish Kumar, Ananthvikas Jayaram, Deenadayalan Munirathnam, Meena Sivasankaran, Revathi Raj, Ramya Uppuluri, Fouzia Na, Biju George, Harsha Prasada Lashkari, Manas Kalra, Anupam Sachdeva, Shishir Seth, Tapas Sabui, Aman Gupta, Karin van Leeuwen, Martin de Boer, Koon Wing Chan, Kohsuke Imai, Osamu Ohara, Shigeaki Nonoyama, Yu Lung Lau, Surjit Singh

**Affiliations:** ^1^ Allergy Immunology Unit, Advanced Pediatrics Centre, Department of Pediatrics, Post Graduate Institute of Medical Education and Research, Chandigarh, India; ^2^ Bone Marrow Transplantation Unit, Department of Internal Medicine, Post Graduate Institute of Medical Education and Research, Chandigarh, India; ^3^ Department of Immunopathology, Post Graduate Institute of Medical Education and Research, Chandigarh, India; ^4^ Department of Immunology, Bai Jerbai Wadia Hospital for Children, Mumbai, India; ^5^ ICMR—National Institute of Immunohaematology, Mumbai, India; ^6^ Department of Pediatrics, Aster CMI Hospital, Bengaluru, India; ^7^ Neuberg Anand Diagnostic and Research Centre, Bengaluru, India; ^8^ Department of Pediatric Hematology and Oncology, Kanchi Kamakoti Child Trust Hospital, Chennai, India; ^9^ Apollo Children’s Hospitals, Chennai, India; ^10^ Christian Medical College, Vellore, India; ^11^ Department of Pediatrics, Kasturba Medical College, Mangalore, India; ^12^ Sir Ganga Ram Hospital, Rajendra Nagar, New Delhi, India; ^13^ Apollo Cancer Institute, Indraprastha Apollo Hospitals, Savita Vihar, New Delhi, India; ^14^ R G Kar Medical College, Kolkata, India; ^15^ Department of Pediatric Rheumatology & Immunology, MEDENS Hospital, Panchkula, India; ^16^ Sanquin Research and Landsteiner Laboratory, Amsterdam Medical Center, University of Amsterdam, Amsterdam, Netherlands; ^17^ Department of Paediatrics and Adolescent Medicine, The University of Hong Kong, Hong Kong, China; ^18^ Department of Pediatrics, National Defense Medical College, Saitama, Japan; ^19^ Department of Community Pediatrics, Perinatal and Maternal Medicine, Tokyo Medical and Dental University, Tokyo, Japan; ^20^ Kazusa DNA Research Institute, Chiba, Japan

**Keywords:** Chronic Granulomatous Disease, India, *Mycobacterium tuberculosis*, Bacillus Calmette Guerin

## Abstract

**Background:**

Chronic granulomatous disease (CGD) is an inherited defect in phagocytic respiratory burst that results in severe and life-threatening infections in affected children. Single center studies from India have shown that proportion of autosomal recessive (AR) CGD is more than that reported from the West. Further, affected patients have high mortality rates due to late referrals and difficulties in accessing appropriate treatment. However, there is lack of multicentric collaborative data on CGD from India.

**Objective:**

To describe infection patterns, immunological, and molecular features of CGD from multiple centers in India.

**Methods:**

A detailed proforma that included clinical and laboratory details was prepared and sent to multiple centers in India that are involved in the care and management of patients with inborn errors of immunity. Twelve centers have provided data which were later pooled together and analyzed.

**Results:**

Of the 236 patients analyzed in our study, X-linked and AR-CGD was seen in 77 and 97, respectively. Male female ratio was 172:64. Median age at onset of symptoms and diagnosis was 8 and 24 months, respectively. Common infections documented include pneumonia (71.6%), lymphadenitis (31.6%), skin and subcutaneous abscess (23.7%), blood-stream infection (13.6%), osteomyelitis (8.6%), liver abscess (7.2%), lung abscess (2.9%), meningoencephalitis (2.5%), splenic abscess (1.7%), and brain abscess (0.9%). Forty-four patients (18.6%) had evidence of mycobacterial infection. Results of molecular assay were available for 141 patients (59.7%)—*CYBB* (44.7%) gene defect was most common, followed by *NCF1* (31.9%), *NCF2* (14.9%), and *CYBA* (8.5%). While *CYBA* variants were documented only in Southern and Western parts of India, a common dinucleotide deletion in *NCF2* (c.835_836delAC) was noted only in North Indian population. Of the 174 patients with available outcome data, 67 (38.5%) had expired. Hematopoietic stem cell transplantation was carried out in 23 patients, and 12 are doing well on follow-up.

**Conclusions:**

In India, proportion of patients with AR-CGD is higher as compared to Western cohorts, though regional differences in types of AR-CGD exist. Clinical profile and mortality rates are similar in both X-linked and AR-CGD. However, this may be a reflection of the fact that milder forms of AR-CGD are probably being missed.

## Introduction

Chronic granulomatous disease (CGD) is an inborn error of immunity (IEI) characterized by a defective respiratory burst in phagocytes, resulting in defective clearance of phagocytosed microorganisms (1). It is caused by mutations in genes encoding different protein subunits of nicotinamide adenine dinucleotide phosphate (NADPH) oxidase enzyme complex (2). Clinical manifestations vary from an immunodeficient phenotype with repeated infections (3, 4) to those characterized by uncontrolled hyper-inflammation (5) and other autoimmune manifestations. Common patterns of infections in CGD include pneumonia, lymphadenitis, hepatosplenomegaly, and abscesses (6).

Laboratory diagnosis of CGD can be made by the nitroblue tetrazolium dye reduction test (NBT) (7, 8) or by flow-cytometry based dihydrorhodamine (DHR) assay (9, 10). The DHR assay is now considered to be the preferred screening test due to its higher reproducibility, sensitivity, rapidity, and ability to detect X linked carriers (11, 12). While several centers in India have published their individual experiences on CGD (13–16), countrywide data have never been collated before. Such collaborative efforts are the need of the hour and are especially important for uncommon conditions like CGD, for which there is paucity of data on disease burden in the country. The present work is the first multi-centric study in India to provide data on clinical, immunological, and molecular features of CGD.

## Methods

We contacted all centers that are recognized as Foundation for Primary Immunodeficiency Diseases (FPID) regional centers for diagnosis or treatment for primary immunodeficiencies in India, and also other Indian institutions involved in care of patients with IEI. All centers were requested to provide clinical and laboratory details of patients with CGD on a pre-designed Excel sheet. Data collection was completed by July 2020. Details included demographic information, family history including consanguinity, clinical manifestations, immunological investigations, genetic diagnosis, treatment, and follow-up.

**Figure 1:**
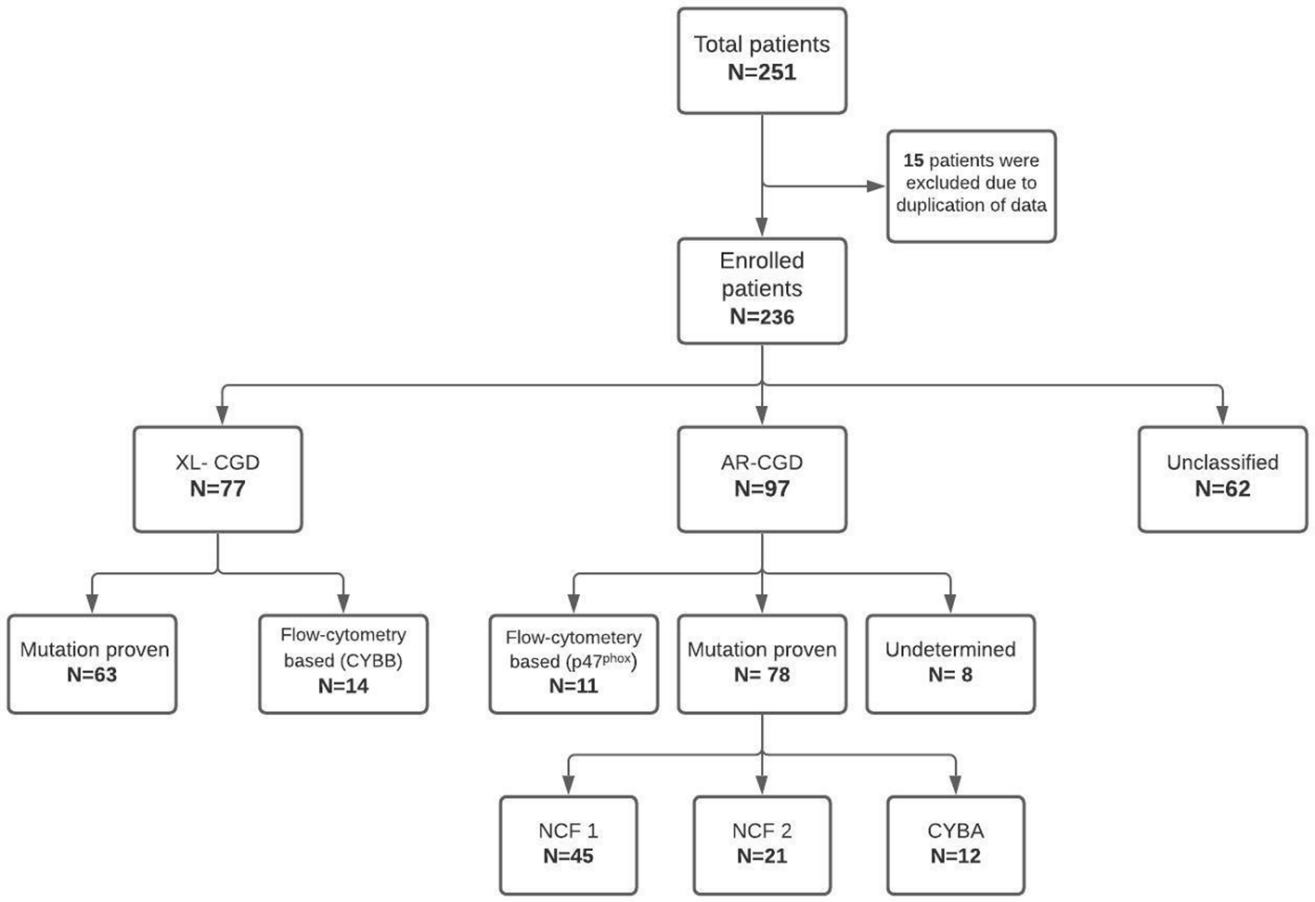
Flowchart showing the classification of patients analyzed in the current study.

Participating institutes included—Postgraduate Institute of Medical Education and Research (PGIMER), Chandigarh, North India (80 patients); Bai Jerbai Wadia Hospital for Children, Mumbai, West India (57 patients); Aster CMI Hospital, Bengaluru, South India (22 patients); National Institute of Immunohematology (NIIH), Mumbai, West India (22 patients); Kanchi Kamakoti Childs Trust Hospital, Chennai, South India (21 patients); Apollo Hospitals, Chennai, South India (17 patients); Christian Medical College, Vellore, South India (five patients); Kasturba Medical College, Mangalore, South-West, India (five patients); Sir Ganga Ram Hospital (SGRH), New Delhi, North India (four patients) and one patient each from R G Kar Medical College, Kolkata, East India; Indraprastha Apollo Hospital, New Delhi, North India; and Medens Hospital, Haryana, North India. Data were collated and subsequently translated on to a database and analyzed (**Figure 1**). Before the analysis, all patient identity details were anonymized. Three (3) female patients had a probable skewed X-linked inactivation. These patients have been described separately.

### Nitroblue Tetrazolium and Dihydro-Rhodamine Tests

Diagnosis of CGD was based on an abnormal granulocyte oxidative burst evaluated by either NBT or flow-cytometry based DHR assay or both. Nitroblue tetrazolium test was available in almost all the centers. However, flow cytometry based DHR was available only in six centers. Analysis of NADPH oxidase components by flow cytometry was carried out only in two centers: National Institute of Immunohematology (NIIH), Mumbai, and Post Graduate Institute of Medical Education and Research (PGIMER), Chandigarh. Details of flow cytometry methods for DHR and NADPH oxidase components at NIIH, Mumbai (13, 14) and PGIMER, Chandigarh (15–17) have been described previously.

### Molecular Assays

Diagnosis was confirmed by molecular analysis in 60% of the patients. At most centers, molecular analysis was carried out at private laboratories (Medgenome Laboratories Pvt. Ltd., India; Strand Genomics Pvt. Ltd., India; Neuberg Anand Diagnostics Pvt. Ltd., India). Illumina platform was used for Next-Generation sequencing (NGS) in these private laboratories with coverage of >80×. Sanger sequencing was used to confirm variants obtained by Next-Generation Sequencing (NGS).

Two centers had in-house facilities for molecular analysis—NIIH, Mumbai and PGIMER, Chandigarh. Molecular assays carried out at NIIH, Mumbai have been detailed in a previous publication (18). Molecular analysis for some patients at PGIMER, Chandigarh (before 2016) was performed at centers in Hong Kong (Department of Pediatrics and Adolescent Medicine, The University of Hong Kong) and Japan (Kazusa DNA Research Institute, Chiba; Tokyo Medical and Dental University, Tokyo; National Defense Medical College, Saitama).

#### Sanger Sequencing and Next-Generation Sequencing at PGIMER, Chandigarh

Genomic DNA was extracted from peripheral blood using DNA extraction kits (QIAamp DNA Blood mini kit, Germany). Depending upon flow-cytometry evaluation for NADPH oxidase component expression, polymerase chain reaction (PCR) was performed for respective genes *i.e. CYBB* (13 exons) for X-linked CGD and *NCF1* (11 Exons), *NCF2* (16 Exons), *CYBA* (six Exons) for AR-CGD. PCR products from genomic DNA were sequenced on an auto fluorescent sequencer (ABI 3500, Applied Biosystems™; Thermo Fisher Scientific, USA) using BigDye™ Terminator (V3.1 Applied Biosystems™). Sequencing primers were the same as those used for PCR. Upon sequencing the results were obtained in.*abi* format and were analyzed using Codon-code aligner for DNA sequence assembly (4.2.5/2013). Patient sequence was compared with reference human sequence obtained from Ensemble database (https://asia.ensembl.org/index.html), and novel variants were further assessed for their disease-causing effect on protein. Prediction tools such as SIFT, PolyPhen-2, Mutation Taster, and CADD score were used to evaluate their pathogenicity. Human Splicing Finder tool was used for splice-site variants.

Since 2018, the center has started performing targeted NGS using a modest 44 gene panel for patients with primary immunodeficiency diseases. Genomic DNA was quantified using Qubit™ Fluorometer (ThermoFisher Scientific, USA) followed by target amplification using PID 2X 2-primer pool panel. Amplified products were partially digested followed by adapter ligation, barcoding, library purification, and amplification. Further, DNA fragments were immobilized on Ion sphere particles and clonally amplified using Ion One Touch™ 2 Instrument. This emulsion PCR results in beads containing amplified and cloned DNA fragment. Elimination of empty beads was carried out using a robotic enrichment system (Ion One Touch™ ES). Finally, the beads containing clonal population of DNA were loaded on to Ion530 Chip. Sequencing was done using Ion S5™ instrument and simultaneously processed on an Ion torrent server for further analysis. Variant calling and analysis were performed using Ion reporter software (ThermoFisher Scientific, USA). The identified variants were validated using Sanger sequencing.

#### Gene-Scan Analysis at PGIMER, Chandigarh

The most common defect in *NCF1* is a dinucleotide deletion, c.75_76delGT at the beginning of exon 2. Conventional Sanger Sequencing or NGS can miss this defect as *NCF1* has two flanking pseudogenes (*ΨNCF1*) with 99% sequence homology with functional gene. So, fluorochrome labeled primers were used to amplify *NCF1* gene and *ΨNCF1* using a method described earlier (Roos et al, 2001). The amplification yielded a mixture of labeled amplicons from the *NCF1* gene and *ΨNCF1* differing by 2 bp in length. This product mixture was analyzed on an Applied Biosystems 3500 Genetic Analyzer using GeneMapper software (Life Technologies, Carlsbad, CA, USA). The amplification product from the pseudogene, being shorter than that from the functional gene, had a shorter retention time and could thus be resolved as distinct peak from the one resulting from the amplicon from the functional gene. Ratio between the two peak heights denotes the relative number of genes and pseudogenes in a test sample.

### Statistical Analysis

Descriptive tests such as medians and ranges were used for continuous variables. Counts and percentages were used for categorical variables. Mann–Whitney U test was used to compare continuous variables between groups. Categorical variables were compared by using the chi-square (x^2^) test or Fisher’s exact test wherever needed. Wilcoxon rank sum test was used to assess efficacy of intervention in a single group. Kaplan–Meier method was used to estimate the survival analysis. Death from any cause was considered as an event, and log-rank test was used to compare the groups. All p values were two-sided and considered significant when p <0.05.

## Results

### Patient Profile

Of the 236 patients, 172 (72.8%) were boys and 64 (27.1%) girls. Details about consanguinity were available for 91 patients, and 40 of them had a history of consanguineous marriage (43.9%). XL-CGD was seen in 77 patients, AR-CGD in 97 children, and the remaining 62 patients could not be categorized as X-linked or AR due to insufficient details of flow cytometry results or molecular analysis (**Figure 1**). AR-CGD was the commonest type in our cohort (XL: AR ratio, 0.8). Three female patients who had skewed X-linked inactivation have been described separately and were not included in the survival analysis.

Median age of onset of initial symptom was 8 months (interquartile range [IQR]: 3–24, range: 0.1 months–20 years), and median age of establishing the diagnosis was 24 months (IQR: 8–60 months, range: 2 weeks–35 years). Median delay in diagnosis was 8 months (IQR: 1.9 months–24 months, range: 0–33.2 years). Follow-up data were available for 98 patients, and median duration of follow-up was 1 year (IQR: 0.1–3 years, range: 1 month–24 years).

### Comparison of Clinical and Laboratory Characteristics Between XL-CGD and AR-CGD

Median age at diagnosis was earlier in XL-CGD than AR-CGD (p = 0.002). However, there was no difference in median age of onset symptoms between these groups (p = 0.074) (**Table 1**). Similarly, median values of stimulation index (SI) derived from DHR assay were also comparable between both groups (p = 0.741) (**Table 1**).

**Table 1:** Comparison of clinical, and laboratory characteristics between XL-CGD, and AR-CGD.

Parameter	XL (n = 77)	AR (n = 97)	p value
Median age at onset of infections (IQR)^a^	6 months (3–20)	12 months (3–35)	0.074
Median age at diagnosis^a^	12 months (7–24)	30 (9–86)	0.002
Median delay in diagnosis of CGD^a^	6 months (1.5–19)	9.2 (1.5–33)	0.353
Median follow-up^a^	1.16 year (1–2.76)	1 year (0.10–2.8)	0.735
Median stimulation index in DHR^a^	1.2 (1–2.76)	1.34 (0.98–2.2)	0.741
Number of patients with >3 infectious episodes^b^	19	15	0.100
Episodes of pneumonia^b^	45	70	0.003
Number of patients with >3 episodes of pneumonia^b^	9	3	0.007
Episodes of superficial abscesses^b^	20	22	0.462
Episodes of deep abscesses^b^	6	7	0.488
Episodes of lymphadenitis^b^	20	27	0.272
Episodes of osteomyelitis^b^	9	9	0.510
Episodes of liver abscesses^b^	6	10	0.500
Episodes of septicemia^b^	8	6	0.540
Number of patients with mycobacterial disease^b^	10	20	0.105
Number of patients with mortality^b^	18	23	1.000
Median Stimulation index <1.5 in DHR^b^	31	44	0.672

^a^Mann–Whitney U test.

^b^Fisher’s exact test or chi-squared test.

Number of episodes of pneumonia was more in AR-CGD (n = 70) than XL-CGD (n = 45) (p = 0.003). Number of patients with >three episodes of pneumonia was also higher in AR-type as compared to XL-CGD (p 0.007). There was no statistical difference between AR-CGD and XL-CGD with regard to following parameters—episodes of superficial abscess (p = 0.462); deep abscess (p = 0.488); lymphadenitis (p = 0.272); and osteomyelitis (p = 0.510). Mortality rate was also comparable in both groups (p = 0.489) (**Table 1**).

On comparing the individual subtypes (*CYBB, NCF1, NCF2*, and *CYBA* defects), age of onset of symptoms and age at diagnosis were earlier in XL-CGD compared to other subtypes (**Table 2**). Mortality rates were low in *NCF1* defect (13.3%) compared to other forms (p 0.040) (**Table 2**).

**Table 2:** Comparison of clinical, and laboratory characteristics between *CYBB, NCF1, NCF2*, and *CYBA* defects.

Parameter	CYBB (n = 63)	NCF1 (n = 45)	NCF2 (n = 21)	CYBA (n = 12)	P value
Median age at onset of infections (IQR)^a^	6 months (3–20)	14.5 months (8–36)	5 months (1–26)	6.5 months (2.2–31.5)	0.037
Median age at diagnosis^a^	12 months (7–30)	36 months (12–96)	21 months (4–90)	30 months (5.5–61.5)	0.027
Median delay in diagnosis of CGD^a^	7 months (1.5–19.5)	12 months (0.7v39)	5.5 months (1.5–26.2)	16 months (2–31.2)	0.781
Median follow-up^a^	1 year (0.1–3.1)	1.3 year (0.1–7.5)	0.5 year (0.01–2)	0.2 year (0.1–5.3)	0.321
Median stimulation index in DHR^a^	1.18 (1–2.09)	1.23 (0–2.01)	1.3 (1.0v2.16)	2.15 (0.29–5.08)	0.626
Number of patients with >3 infectious episodes^b^	19 (30.1%)	7 (15.5%)	6 (28.5%)	0	0.051
Episodes of pneumonia^b^	41 (65%)	32 (71.1%)	16 (76.1%)	8 (66.6%)	0.012
Number of patients with >3 episodes of pneumonia^b^	9 (14.2%)	3 (6.6%)	0	0	0.058
Episodes of superficial abscesses^b^	17 (26.9%)	8 (17.7%)	10 (47.6%)	0	0.343
Episodes of deep abscesses^b^	5 (7.9%)	1 (2.2%)	1 (4.7%)	2 (16.6%)	0.825
Episodes of lymphadenitis^b^	19 (30.1%)	13 (28.8%)	7 (33.3%)	2 (16.6%)	0.608
Episodes of liver abscesses^b^	6 (9.5%)	4 (8.8%)	4 (19%)	0	0.408
Episodes of septicemia^b^	8 (12.6%)	6 (13.3%)	3 (14.2%)	0	0.145
Number of patients with Mycobacterial disease^b^	8 (12.6%)	10 (22.2%)	4 (19%)	3 (25%)	0.144
Number of patients with mortality^b^	17 (26.9%)	6 (13.3%)	8 (38%)	6 (50%)	0.042
Number of patients with stimulation index <1.5 in DHR^b^	28 (44.4%)	18 (40%)	12 (57.1%)	3 (25%)	0.462

^a^Mann–Whitney U test.

^b^Fisher’s exact test or chi-squared test.

### Clinical Characteristics of p47^phox^ Defect in Comparison to p67^phox^- Defect

Median age at symptom onset was higher in p47^phox^ defect (14 months) when compared to p67^phox^ defect (5 months). The difference was, however, not statistically significant. Median age at diagnosis and median delay in diagnosis was comparable between two groups (**Supplementary Table 1**). There was also no statistical difference between p47^phox^ and p67^phox^ deficiency with regard to the following parameters: number of patients with >three infectious episodes (p 0.326); number of patients with more than three episodes of pneumonia (p = 0.545); and colitis (p 0.441) (**Supplementary Table 1**).

### Infection Profile

We document 559 episodes of infections in 236 patients over 298.6 patient–years of follow-up (**Table 1**). Median number of episodes of infections was two (IQR 1–3 episodes).

#### Localization of Infection

##### Lung

Pneumonia was seen in 169 patients (71.6%), and the total number of episodes of pneumonia was 242. Due to indolent presentation of pneumonia in CGD, and India being an endemic country for tuberculosis, several patients with difficult to treat pneumonia were empirically started on antitubercular therapy (ATT) before the diagnosis of CGD could be ascertained. Fifty-three (54%) of 96 patients with persistent pneumonia had received empirical ATT in our cohort. Number of ATT courses ranged from one to three. Three patients with CGD had extensive bronchiectasis with pulmonary hypertension at time of diagnosis—all three had received multiple courses of empirical ATT for many months before the diagnosis of CGD was confirmed in them.

In our cohort, fungal pneumonia was commonest (26.8%), followed by bacterial (23.9%) and mycobacterial infections (11.5%) (**Table 3**). The commonest organisms were *Aspergillus* sp., *Mycobacterium* sp., *Staphylococcus aureus*, *Pseudomonas* sp., *Klebsiella* sp., and *B. cepacia* (**Table 2**). Confirmed fungal etiology either on lung histopathology or on microbiological cultures from affected tissues was documented in 18 of 62 episodes of suspected fungal infection. The remaining patients also probably had a fungal infection as they had positivity for biomarkers such as galactomannan and/or beta-D-glucan. Twenty patients (8.4%) required mechanical ventilation due to severe lung disease or associated septicemia. Other pulmonary complications include lung abscesses (n = 7; 2.9%), bronchiectasis (n = 5; 2.1%), and contiguous rib osteomyelitis (n = 5; 2.1%).

**Table 3:** Profile of microorganisms in patients with pneumonia.

Microorganisms	Number of episodes (n = 242)		%
**Bacterial organisms**	90		37.1
• *Staphylococcus aureus*	14		5.7
• Mycobacterial disease	28		11.5
• *Mycobacterium tuberculosis*	24		9.9
• Disseminated BCG	4		1.6
• *Pseudomonas* sp.^#^	11		4.5
• *Klebsiella pneumonia*	8		3.3
• *Burkholderia cepacia*	8		3.3
• CONS	9		3.7
• *Acinetobacter* sp.	3		1.2
• *Nocardia* sp.	2		0.8
• Others*	5		2
**Fungal organisms**	63		26
	**Probable**	**Proven**	
• *Aspergillus* sp.	38	10	19.8
• *Aspergillus* sp. (subspecies: not known)		4	1.6
• *A. fumigatus*		4	1.2
• *A. flavus*		2	0.8
• *Candida* sp. (subspecies: not known)	9	1	4.1
• *Candida tropicalis*		1	0.4
• *Candida lusitanie*		1	0.4
• *Fusairum dimerium*	–	1	0.4
• *Mucor* sp.		2	0.8

*Streptococci sp. (n=2); Chryseobacterium gleum (n=1); Fransciella noatuensis (n=1); Proteus mirabilis (n=1).

CONS, Coagulase Negative Staphylococcus sp.^#^Pseudomonas aeruginosa (n=6).

Mycobacterial infections were documented in 44 patients (18.6%)—*Mycobacterium tuberculosis* in 25, Bacillus Calmette Guerin (BCG) related complications in 17 (localized disease in 13, and disseminated disease in four), and infection due to non-tuberculous mycobacteria in two (**Table 4**). Two patients with disseminated tuberculosis had a stormy course—one patient had anterior chest wall cold abscess, hepatosplenomegaly, and computed tomography (CT) guided lung biopsy showed granulation tissue with AFB positivity; one patient presented with pericardial and pleural effusions. Both of them also had lymphadenopathy and rib osteomyelitis at time of diagnosis. While one improved after initiation of conventional ATT, the other succumbed to the illness due to concomitant *Mucorales* infection in the lung.

**Table 4:** Spectrum of mycobacterial disease in our cohort.

Mycobacterial disease	No of patients (Organism positive)(n = 44)	%
***Mycobacterium* sp.**	27 (2, non-tuberculous)	61.3
• Pneumonia	24	54.5
• Lymphadenitis	6	13.6
• Abdominal	2	4.5
• Osteomyelitis	2	4.5
• CNS	1	2.2
• Skin (Lupus vulgaris)	1	2.2
• Disseminated tuberculosis	9	20.4
**BCG infection**	17	38.6
• Localized BCG adenitis	13	29.5
• Disseminated BCG	4	9

##### Blood-Stream Infections

Though patients with CGD usually do not have a proclivity for dissemination of infection, 21 patents (9%) in our cohort had septicemia. Blood-stream infections (BSI) were found in 75 of 551 infectious episodes (**Table 4**). Bacterial infections were commonest (70/75 episodes, 93.3%). Some of the signature organisms that were isolated included *S. aureus* (21.3%); *Burkholderia cepacia* (13.3%); *Pseudomonas* sp. (9.3%); *Klebsiella* sp. (8%); followed by other organisms (**Table 5**).

**Table 5:** Isolation of microorganisms in blood stream infections.

Microorganisms	No of episodes isolated (n = 75)	%
**Bacteria**	70	93.3
• *Staphylococcus aureus*	16	21.3
• *Klebsiella pneumoniae*	6	8
• *Pseudomonas* sp.^#^	7	9.3
• *Burkholderia* sp.^	10	13.3
• CONS	7	9.3
• *Entercoccus* sp.	4	5.3
• *Salmonella* sp.^##^	6	8
• *Acinetobacter* sp.^^^^	5	6.6
• Others*	7	9.3
• Gram negative septicemia	2	2.6
**Fungal**	5	6.6
• *Aspergillus fumigatus*	2	2.6
• *Candida* sp.	2	2.6
• *Candida tropicalis*	1	1.3

*Streptococci sp. (n=2); E. coli (n=2); Bacillus subtilis (n=1); Neisseria meningitidis (n=1);

Citrobacter freundi (n=1); Fransicella sp. (n=1).

CONS, Coagulase Negative Staphylococcus sp.

^#^Pseudomonas aeruginosa (n=3); Pseudomonas stutzeri (n=2). ^Burkholderia cepacia (n=3); B. cenocepacia (n=1).

^##^Non-typhoidal Salmonella.

^^^^Acinetobacter baumanni (n=3).

##### Lymphadenitis

Lymphadenitis was seen in 74/236 patients (31.6%), and 94 episodes were documented in our cohort. Microorganisms were isolated from lymph node aspirate in 25/74 patients (33.7%). The commonest organisms isolated were *Staphylococcus* sp. (40%), *Mycobacterium* sp. infections (36%) followed by *Burkholderia* sp., *Klebsiella* sp., and *Pseudomonas* sp. (**Table 6**). Two patients underwent surgical resection for recurrent lymphadenitis that was recalcitrant to medical therapy.

**Table 6:** Organism profile in lymphadenitis.

Microorganisms	Number of events (n = 25)	%
**Bacteria**	24	96
• *Staphylococcus aureus*	10	40
• Mycobacterial infection	9	36
• *M. tuberculosis*	2	8
• BCG adenitis	7	28
• *Bulkholderia cepacia*	3	12
• *Klebsiella pneumoniae*	1	4
• *Pseudomonas aeruginosa*	1	4
**Fungal**		
• *Candida parapsilosis*	1	4

##### Skin and Subcutaneous Abscesses

Skin and subcutaneous abscesses were found in 56 patients (23.7%), and a microorganism could be isolated from pus culture in 17/56 patients (30.3%) (**Table 7**). Majority (88.2%) were due to bacterial infections and among these, *S. aureus* was isolated in eight patients (53.3%). Perianal abscesses were found in six patients (2.5%) with CGD.

**Table 7:** Spectrum of microorganisms in patients with skin and subcutaneous abscesses.

Microorganisms	Number of patients (n = 56)	%
Bacteria	**15**	**26.7**
• *Staphylococcus aureus*	8	14.2
• *Klebsiella pneumoniae*	2	3.5
• *Pseudomonas* sp.*	3	5.3
• *Burkholderia cepacia*	2	3.5
• *Acinetobacter baumanii*	1	1.7
Fungal	**2**	**3.5**
• *Candida* sp.	1	1.7
• *Basidiobolus* sp.	1	1.7

*Pseudomonas aeruginosa (n=2).

##### Liver Abscess

Seventeen patients presented with liver abscesses (7.2%), and one among these had a recurrent liver abscess (19). Four patients with liver abscess had concurrent other site abscesses (staphylococcal skin pustules in one patient, *Pseudomonas aeruginosa* cervical abscess in one patient, and multiple deep-seated abscesses noted in two other patients).

*S. aureus* was isolated from pus in 5/17 patients (29.4%). Three patients with liver abscess were administered corticosteroids besides intravenous antibiotics. Rupture of liver abscess warranting surgical intervention was documented in one child who had not received corticosteroids.

##### Other Deep-Seated Abscesses

Seven patients had lung abscess—*Nocardia* sp. and *Aspergillus* sp. was isolated in one patient each. Splenic abscess was found in four patients. Two patients developed brain abscesses, and *Aspergillus fumigatus* was isolated from the pus in both. One child also had retropharyngeal and parapharyngeal abscess.

##### Bone

Infective osteomyelitis was found in 20 patients (8.6%). Site of involvement was variable: ribs—five; lower extremities [tibia (n = 2), fibula (n= 1)]; vertebrae—one; radius—one; skull—one; and small bone osteomyelitis of the hands in two patients. *Aspergillus fumigatus* was isolated from four patients; *Enterobacter* sp., *Chromobacterium violaceum*, *A. terreus*, and *Serratia marcescens* were isolated in one patient each (16, 19, 20). Mycobacterial infection was responsible for two cases of osteomyelitis (one with *M. tuberculosis*, and another with atypical mycobacterial disease). Surgical resection of ribs was required in two patients, while other patients were managed conservatively.

##### Other Sites

Meningoencephalitis was seen in six patients. Two children had complicated meningitis with hydrocephalus. *Aspergillus nidulans* was isolated from CSF from one of them. Ten children had otitis media (4.2%). Urinary tract infection (UTI) was seen in 12 patients (5.1%).

### Non-Infectious Manifestations

Twelve patients (5.1%) had colitis. Twenty-three episodes of colitis were noted in the cohort, and one patient had had six relapses. One child had been diagnosed to have inflammatory bowel disease elsewhere. He had received infliximab for recalcitrant colitis before the diagnosis of CGD and had developed severe pneumonia following the therapy. Glucocorticoids, mesalamine, and azathioprine were used in seven, five, and three patients respectively. A child with XL-CGD had complete remission of colitis following hematopoietic stem cell transplantation (HSCT).

Lung granuloma was documented in 16 patients (6.8%), and liver granuloma in two patients. Secondary hemophagocytic lymphohistiocytosis (HLH) was documented in six patients (2.5%) (21). Common infective triggers identified were blood stream septicaemia (n = 3) [*Francisella noatuensis* (one), *Burkholderia cenocepacia* (one), *Candida albicans* (one)], pneumonia (n = 4) [*Nocardia* sp. (n = 1), probable *Aspergillus* sp. (n = 1)], and disseminated BCGosis (n = 1) (21). Other non-infective manifestations found in our cohort included chilblains (n = 2), HLA-B27 related arthritis (n = 1), Kawasaki disease with coronary artery aneurysm (n = 1), unexplained chronic kidney disease (n = 1), and intestinal obstruction in one patient.

### Molecular Diagnosis

**Figure 2:**
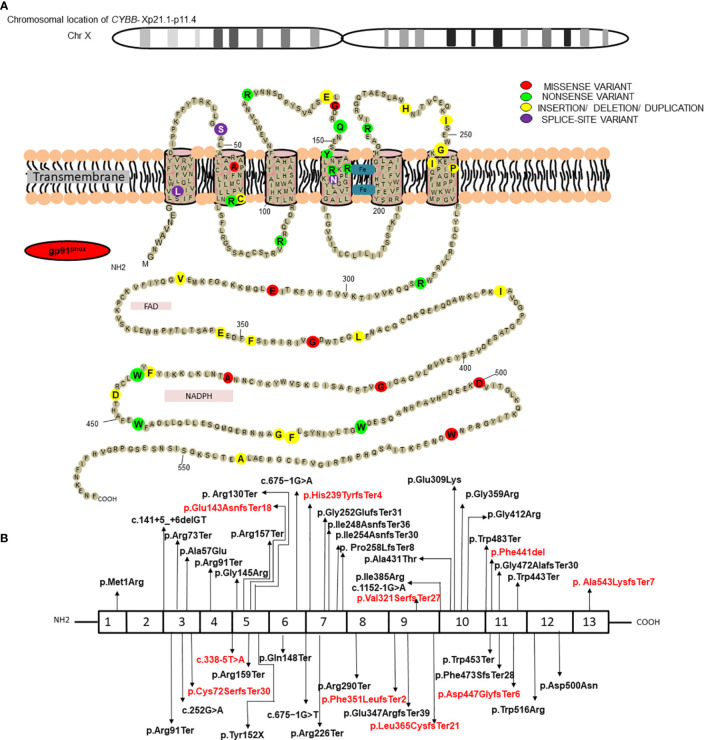
**(A)** Position of protein change in gp91^phox^ for the molecular variants in *CYBB* identified in current study; **(B)** Molecular variants identified in different exons of *CYBB* and corresponding protein domains. *Novel variants have been highlighted in red*.

**Figure 3:**
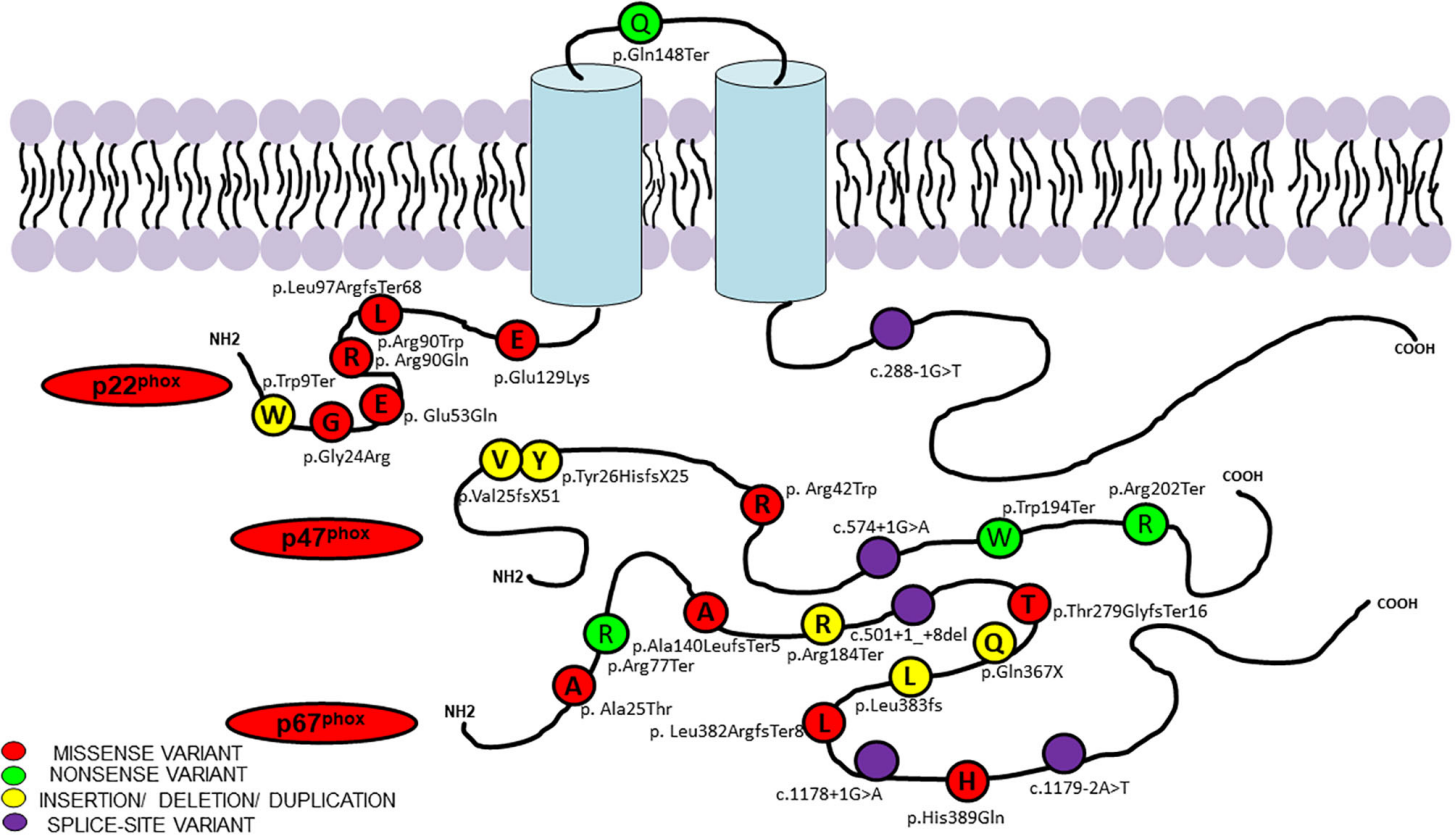
Molecular variants and position of protein change in p47^phox^, p67^phox^, and p22^phox^ identified in current study.

**Figure 4:**
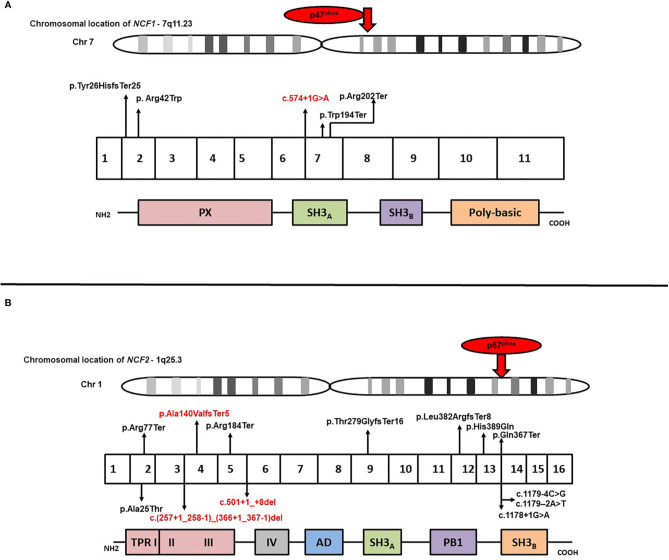
**(A)** Molecular variants identified in different exons of *NCF1* and corresponding protein domains; **(B)** Molecular variants identified in different exons of *NCF2* and corresponding protein domains. *Novel variants have been highlighted in red*.

Results of genetic analysis were available for 141 patients (59.7%). Pathogenic variants in the *CYBB* were the most common (63/141; 44.7%), followed by *NCF1* gene variants (45/141; 31.9%) whereas genetic variants in *NCF2* and *CYBA* comprised 14.9% (21/142) and 8.5% (12/141) of all the variants, respectively (**Supplementary Table 2**) (22–30). Fifteen of the 141 genetic variants were novel; 10 in the *CYBB* gene, three in the *NCF2* gene, and two in the *NCF1* gene. Nonsense variants comprised majority of *CYBB* gene variants (21/63; 33.3%), followed by missense variants (11/63; 17.4%), splice-site variants (10/63; 15.8%), deletions (9/63; 14.3%), insertions (5/63; 7.9%), duplications (4/63; 6.3%), and promoter-site variant (n = 1) (**Figure 2**). Five patients had a previously reported synonymous variant c.252G>A; p.A84(=) which is located proximal to the splice donor site of intron 3 and results in skipping of exon 3 of *CYBB*. Most patients with autosomal recessive CGD due to gene variants in the *NCF1* gene had the common dinucleotide deletion in exon 2 of the gene. However, variants were also detected in other exons of the gene including a large deletion involving exons 2–10 of the *NCF1* gene and nonsense variants in exon 7 in two other patients (**Figures 3**, **4**). *NCF2* gene variants were more common in patients from North India (15/22; 68.1%) compared to patients from West or South India (7/22; 31.9%). Majority of the patients with *NCF2* gene variants from North India (10/15; 66.7%) had a common dinucleotide deletion in exon 9 (c.835_836delAC; p.Thr279GlyTer16) (31) of the gene resulting in alteration of the reading frame and termination (**Figures 3**, **4**). *CYBA* gene variants in contrast were exclusively seen in patients from South and West India. Most of the *CYBA* gene variants were located in exon 4 of the gene (6/12; 50%) and one patient had a large deletion involving exons 2–4 of the *CYBA* gene (**Figure 3**).

Three X-linked carriers of *CYBB* defect had manifestations of CGD in form of severe infections probably as a result of skewed inactivation (32, 33). A carrier of X-linked CGD developed manifestations of severe lupus malar rash, arthritis, positivity for anti-nuclear and anti-double stranded DNA antibodies, seizures, and demyelinating lesions in the brain and spinal cord (21). She ultimately succumbed to her illness.

### Treatment

All patients were started on prophylactic antimicrobials, usually a combination of cotrimoxazole and itraconazole. Data on breakthrough infections after initiation of antimicrobial prophylaxis were available for 78 children. Statistical analysis showed a significant reduction in episodes of pneumonia; lymphadenitis (p = 0.003); skin, and subcutaneous abscesses (p < 0.001); deep-seated abscesses (p = 0.007); osteomyelitis (p = 0.035); liver abscess (p = 0.02); and septicemia (p = 0.007) in those compliant to antimicrobials (**Table 8**). Corticosteroids were used in four children with liver abscess, two patients with pneumonia, and in two patients with HLH.

**Table 8:** Comparison of infectious episodes before and after prophylactic therapy in 78 patients.

Pattern of infections	Pre-diagnosisn = 78	Post-diagnosisn = 78	p value*
Pneumonia	115	25	0.001
Lymphadenitis	37	10	0.003
Skin and subcutaneous abscess	32	2	0.001
Osteomyelitis	9	2	0.035
Deep abscesses (excluding liver abscess)	15	0	0.007
Liver abscess	7	1	0.020
Cellulitis	2	0	0.157
Septicemia	9	1	0.007
Otitis Media	4	1	0.180
Diarrhea	4	0	0.157

*Fisher’s exact test or chi-squared test.

Twenty-three patients (9.7%) in the present cohort underwent an HSCT [X-linked—12; AR—three (one each in *NCF1*, *NCF2*, and *CYBA*); unclassified—eight]. Eight patients underwent transplantation with matched related donors. Haploidentical transplantations were carried out in seven patients, while matched unrelated transplantations were performed in three patients, and umbilical cord blood transplantation in one patient. Acute graft *versus* host disease (GVHD) occurred in seven of 23 transplant recipients, and two patients succumbed to acute GVHD. Complete donor chimerism was attained in 16/23 recipients (68.1%). Six patients succumbed to transplant related complications (two patients had GVHD; one patient had primary graft failure; and one patient had CNS complications).

### Survival Analysis

**Figure 5:**
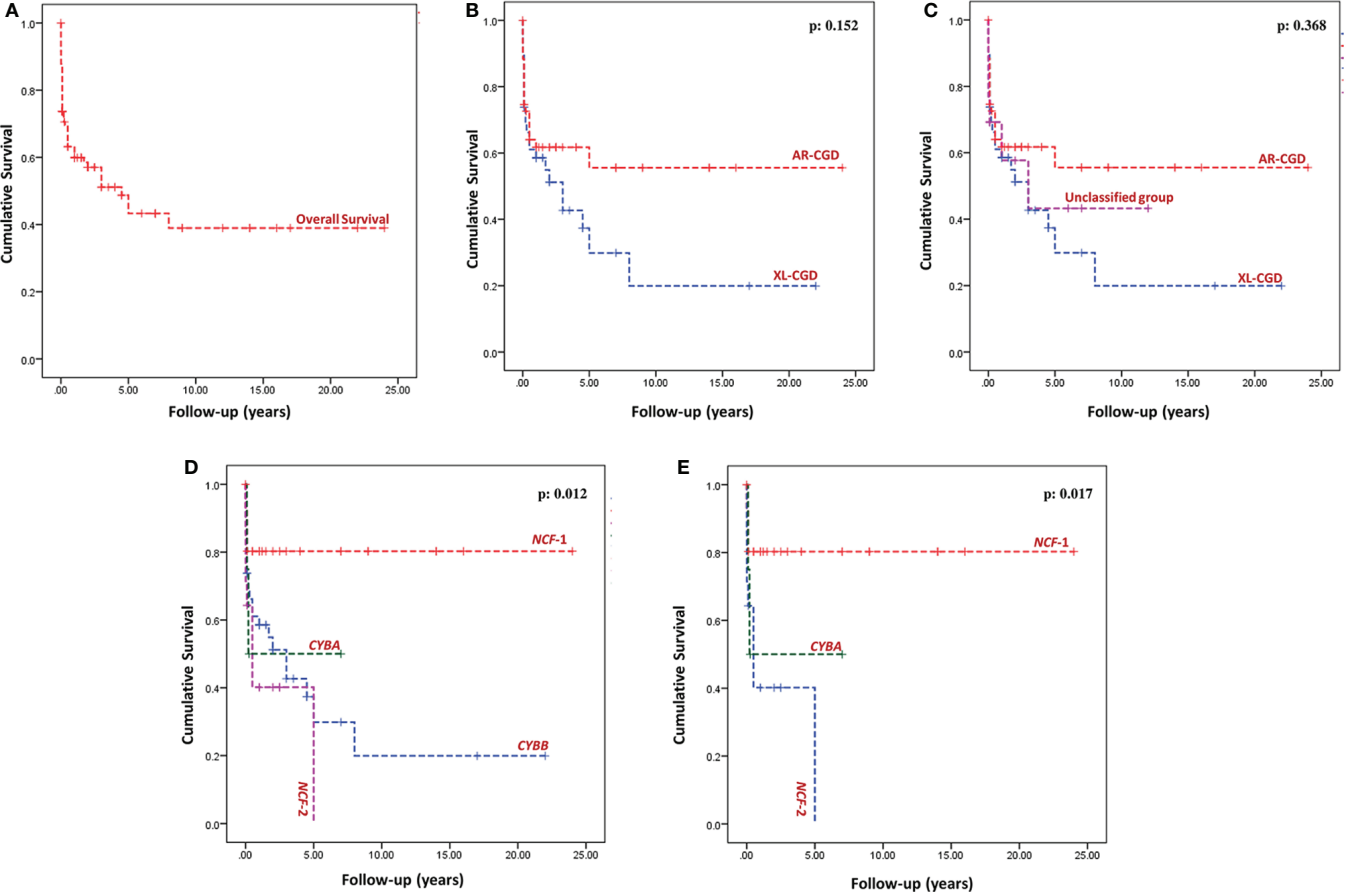
Kaplan–Meier survival curves—**(A)** Overall survival of the entire cohort; **(B)** Comparison of overall survival between AR-CGD and XL-CGD, p = 0.152 (Log Rank Mantel–Cox); **(C)** Comparison of overall survival between Unclassified group, AR-CGD and XL-CGD, p = 0.368 (Log Rank Mantel-Cox); **(D)** Comparison of overall survival between individual subtypes (*CYBB, NCF1, NCF2*, and *CYBA*), p = 0.012 (Log Rank Mantel–Cox); **(E)** Comparison of overall survival between *NCF1, NCF2*, and *CYBA* defects p = 0.017 (Log Rank Mantel–Cox).

Outcome details were available for 174 patients (73.7%), and follow-up duration was available for 98 patients (292.6 patient-years of follow-up). Sixty-seven of 174 patients (38.5%) had expired at the time of analysis (XL-CGD: 27; AR-CGD: 28; and undetermined: 12) (**Figure 5A**). The cumulative survival of XL-CGD and AR-CGD was comparable on Kaplan–Meier analysis (p: 0.152) (**Figures 5B, C** **)**. Cumulative survival comparisons between individual subtypes (*CYBB, NCF1, NCF2*, and *CYBA*) showed better survival in NCF1 defect compared to other subtypes (p 0.012) (**Figures 5D, E** **)**.

Median age at diagnosis in individuals who succumbed to illness was 14 months (IQR 7–40 months), and the median age at time of death was 19 months (IQR 7.8–53.6 months). All patients were under antibiotic prophylaxis. Details of terminal events were available only for 37 out of the 61 patients who did not undergo HSCT. Terminal events that were observed include pneumonia in 30 patients (81%); septicemia (10 patients, 27%); brain abscess (two patients, 5.4); lung abscess, liver abscess, and meningitis in one patient each (2.7%). Three patients (8.1%) also had concomitant HLH at terminal illness.

Details of microbiological profile were available in 22 of 37 patients (59.4%). *Aspergillus* sp. was isolated in eight patients (26.6%) including A. *fumigatus* in two patients and A. *flavus* in one patient; *M. tuberculosis*, Mucorales, *Nocardia* sp. in two patients each (6.6%); *Chryseobacterium gleum*, *Pseudomonas aeruginosa*, *Klebsiella pneumoniae*, *Candida lusitanea*, and cytomegalovirus were isolated in one patient each (3.3%). Out of 10 patients with septicemia, microorganisms could be isolated in eight patients [*S. aureus* (two), *Candida* sp. (one), *C. tropicalis* (one), *P. aeruginosa* (one), *P. stutzeri* (one), *Acinetobacter* sp. (one), *B. cepacia* (one), *B. cenocepacia* (one), and *K. pneumoniae* (one)]. *Aspergillus fumigatus* was isolated in a patient with brain abscesses, and *A. nidulans* was isolated in cerebrospinal fluid in a patient who succumbed to meningitis.

## Discussion

CGD was first described in four male children from Minnesota in the year 1954 (34) and was later referred as ‘fatal granulomatosus of childhood’ by Berendes et al. in 1957 (35). Subsequently, molecular mechanism of disease pathogenesis (36) and genetic defect (*CYBB*) was discovered in the years 1967 and 1986 (37, 38), respectively. CGD is now one of the commonly recognized IEI in children. Prognosis of affected children has improved significantly over the last few decades with the introduction of long-term antimicrobial prophylaxis and initiation of curative therapies such as HSCT. Incidence of CGD differs between populations, and it ranges from an estimated one per 200,000 lakh live births in the US and Europe (1, 6), to 1.5 per 100,000 live births among Arabs in Israel where consanguinity rates are high (39).

Initial reports of CGD from India date back to late 1990s (40). Subsequently, the two Indian Council of Medical Research Centres for Advanced Research in Primary Immunodeficiency Diseases (PGIMER, Chandigarh and NIIH, Mumbai) published large case series on CGD from their respective centres (13, 15, 16). Several case reports and case series from other centers have also emanated in the last few years. The present multi-centric study is an effort to collate the data of patients with CGD diagnosed at multiple centers across the country to understand epidemiology, profile of infections, and molecular spectrum of CGD in India. For this study, we obtained data on 236 patients with CGD diagnosed at 12 different centers in India.

We observed a higher proportion of AR forms of CGD as compared to X-linked forms. This is consistent with the previously published data on CGD from India (15). Higher proportion of AR-CGD may be a reflection of high rates of consanguinity and endogamous marriages in the country. Median age at diagnosis in our cohort was 24 months (IQR: 8–60 months), which is comparable with data from other large multi-centric studies from the USA, Europe, and China (1, 6, 41, 42) (**Supplementary Table 3**). Patients with X-linked CGD have an earlier age of presentation and are reported to have a more severe illness as compared to patients with AR-CGD. We also observed that age at onset of infections and diagnosis of AR-CGD was significantly higher than XL-CGD. However, unlike reports from several other countries (1, 43), there was no difference in survival and mortality rates between AR and XL types of CGD. We attribute this difference to three factors—delays in diagnosis of patients with AR-CGD, a probable missed diagnosis of milder forms of AR-CGD in many patients probably due to lack of awareness especially among the adult physicians, and lack of appropriate laboratory facilities for diagnosis of CGD at many hospitals in India. At present, NBT test can be performed at most centers involved in care of IEI and DHR is currently performed only at six centers. However, many tertiary care hospitals in the country still do not have the facility to perform either NBT or DHR. Median follow-up duration of our cohort is 1 year suggesting that majority of patients are only diagnosed only in the recent years. Increase in awareness of IEI and upscaling of laboratory facilities for carrying out basic immunological investigations are the needs of the hour.

Among the breakthrough infections, pneumonia was most common followed by abscesses (skin, subcutaneous, and deep abscesses, inclusive of liver abscesses), lymphadenitis, and BSI. India, being a country endemic for tuberculosis, it is not surprising that many children with persistent pneumonia (54%) in our cohort had received empirical ATT in our cohort. This has led to significant delays in diagnosis of CGD. Pediatricians and internists practising in developing countries such as India need to keep in mind that persistent pneumonia may be a presentation of CGD. Empirical ATT may not be warranted in such cases.

*Aspergillus* sp. is the most common etiology for pneumonia in our cohort followed by gram-negative bacteria, *Mycobacterium* sp., and *Staphylococcus* sp. Microbiological profile of pneumonia is similar to cohorts from China (44), Iran (45), and Turkey (43). Pneumonia spreading to contiguous tissues such as ribs and vertebra has been documented only with pneumonia due to fungal or mycobacterial etiology (Aspergillus sp.—four; *Mucorales* sp. —one; *Mycobacterium* sp. —two). Pulmonary infection due to *Mucor* sp. was documented in two patients who had not been exposed to corticosteroids. Delays in diagnosis, malnutrition, and prolonged antibiotic therapy for pneumonia could be the reasons for the development of *Mucorales* infection in our cohort. *Nocardia* sp. has been isolated in two patients—one with a lung abscess and the other with persistent pneumonia. Unusual bacteria documented with pneumonia include *C. gleum*, *Citrobacter* sp., and *Francisella noatuensis*. A patient with persistent pneumonia had grown *C. lusitaniae* from the lung tissue which was resistant to amphotericin B.

A wide spectrum of microorganisms that have been isolated in CGD patients with pneumonia suggests the importance of microbiological isolation of organism and appropriate targeting of anti-microbials. We suggest that patients with severe pneumonia in CGD must be managed in tertiary-care centers with facilities for advanced microbiological testing such as MALDI-TOF, 16S rRNA PCR, fungal cultures, and PCR.

Apart from signature organisms such as *Aspergillus* sp. and *Staphylococcus* sp., we also documented a high incidence of mycobacterial infections (18.5%) in our cohort. Most common presentation of mycobacterial infection was pneumonia followed by disseminated forms and lymphadenitis (**Table 4**). Localized or disseminated infections due to BCG have been documented in 8% of patients in our cohort. Abnormal BCG response has been documented at higher rates in other cohorts such as Iran (55.9%) (45), Turkey (22.5%) (43), Latin America (29.6%) (46), and China (64%) (41) that also administer BCG to all children. We do not investigate all patients with BCG adenitis for CGD. It is possible that milder forms of CGD may present only as BCG adenitis and that such patients may have been missed in our cohort. Incidence of *Serratia* infection in our cohort is very low (2/236, 0.8%) when compared to studies from North America and Europe (47). Possible reasons for this observation include tropical climate patterns and low microbiological isolation rates. Lee et al. have previously reported that infection due to *Burkholderia pseudomallei* and *Chromobacterium violaceum* in CGD have been predominantly noted in tropical countries (48). Melioidosis is common in Southern India, and we have also documented melioidosis in a patient from South India who had X-linked CGD (49). Septicemia due to *C. violaceum* has also been documented in a child with *NCF1* defect who hailed from Central India. It is possible that many patients with melioidosis are not screened for CGD in the country and diagnosis of CGD may be missed in them.

*Staphylococcus aureus* was the commonest organism isolated in suppurative lymphadenitis and skin abscess. Other bacteria isolated in such infections included gram-negative organisms like *Burkholderia* sp., *Klebsiella* sp., and *Pseudomonas* sp. Fungi such as *Candida* sp. and *Basidiobolus* sp. were only occasionally isolated in such infections. The microbiological spectrum in these infections is similar to reports from other countries (1, 6, 43, 50). Among the BSI, the microbiological spectrum was almost similar—*Staphylococcus* sp. was the commonest, followed by *Burkholderia* sp. and other gram-negative bacteria. However, non-typhoidal *Salmonella* (8%) was isolated more frequently from blood as compared to other sites. This suggests the importance of frequent handwashing and avoidance of ingestion of raw food items in patients with CGD, as these factors increase the risk of acquiring non-typhoidal *Salmonella*. *S. aureus* is the only organism documented in patients with liver abscess. Oral prednisolone was successfully used in three children with liver abscess for enhanced resolution, and none of them required surgical intervention. Our experience is in line with recent evidence concerning use of corticosteroids in liver abscess in CGD (51).

Common non-infective manifestations documented in our cohort include inflammatory bowel disease-like colitis, visceral granuloma, and secondary HLH. We document a lower rate of colitis (5%) in our cohort compared to studies from North America, Europe and China. However, our rates are comparable to cohorts from Turkey, Iran, and Israel, where autosomal recessive forms of CGD are predominant compared to X-linked CGD (**Supplementary Table 3**) (43, 45). Two patients who had colitis since early infancy were managed as inflammatory bowel disease elsewhere, and were diagnosed to have CGD at 5 and 7 years, respectively. One of the children also developed fulminant pneumonia following infliximab therapy for IBD-like colitis, similar to the report by Uzel et al. (52). This suggests that work-up for CGD must be considered in children with early-onset colitis – pediatricians and gastroenterologists need to be aware of this presentation. Unlike reports from other studies (1, 53), children with AR-CGD had a higher incidence of colitis in our cohort compared to X-linked forms. The difference is probably due to predominance of AR forms of CGD in the present series. While four patients achieved remission with only oral prednisolone and mesalamine, three achieved partial control with azathioprine. Complete remission of colitis following HSCT was documented in a child with X-linked CGD. This shows that immunological reconstitution following HSCT is also beneficial for the inflammatory component of CGD.

Flow cytometry-based evaluation of NADPH oxidase components is a surrogate marker for identifying the genetic defect in CGD. However, this assay can be currently performed in only in two centers in India—PGIMER, Chandigarh and NIIH, Mumbai. We identified regional heterogeneity in molecular spectrum of CGD within India. Overall, *NCF1* is the commonest molecular defect identified in patients with AR-CGD, similar to the cohorts from other parts of world. However, *CYBA* is the second common type of AR-CGD in patients from Southern and Western India, whereas, *NCF2* defect is commoner in patients from North India. Moreover, a common dinucleotide deletion in *NCF2* (c.835_836delAC) was observed in 10 patients from North India, suggesting a Founder effect in this population. Observed regional differences in molecular spectrum could be due to heterogeneity in genetic background of the population in India. Genescan is considered to be the preferred mode for identification of common dinucleotide deletion in Exon 2 of *NCF1* (c.75_76delGT). This modality is, however, currently available only at Chandigarh and Mumbai. The preferred molecular approach for work-up of patients with CGD at Mumbai has been detailed in a previous publication. At Chandigarh, we follow a similar approach, except that Sanger sequencing for *NCF2* c.835_836delAC variant is carried out first for patients who have decreased p67^phox^ expression by flow cytometry, as this variant is commonly and exclusively observed in North Indian population. This is followed by NGS whenever required. We have not identified p40^phox^ (*NCF4*) defect in our cohort. However, patients with *NCF4* defect can have near normal neutrophil stimulation in the conventional DHR assay done using phorbol myristate acetate (PMA) as the stimulant (54). It is possible that this defect has been missed in many patients in India, as all patients suspected to have CGD were screened with DHR assay using stimulation with PMA. Recently, *EROS* (*CYBC1*) defect resulting in CGD has also been described (55). We have not screened for this defect in our cohort.

Most of the patients in our cohort were kept on antimicrobial prophylaxis—cotrimoxazole and itraconazole. Interferon-gamma was not used in any of our patients as it is not available in India. Number and severity of infections, especially those due to *Staphylococcus* sp., significantly came down after initiation of antimicrobial prophylaxis (**Table 8**). However, many patients had documented breakthrough infections even while being continued on prophylactic medications. Overall mortality in our cohort was 38.5% which is similar to reports from other countries such as Mexico and China (41, 50, 56, 57). Our mortality rates are, however, much higher than those reported from the USA, Iran, and European countries. Delays in diagnosis, severe infection at the presentation, and lack of widespread availability of pediatric HSCT services in the country accounted for high mortality rates in our cohort. However, we also document significant decrease in number of infections following initiation of antibiotic prophylaxis (**Table 8**). This suggests that patients with CGD in developing countries, especially AR forms (*NCF1* defect), can have a reasonable quality of life provided they are diagnosed early and continued on long-term antimicrobial prophylaxis (**Figure 5**).

HSCT could be carried out in only 23 patients in our cohort. Most of the HSCTs have been carried out in Apollo Children’s Hospitals, Chennai. Only five other centers (BJ Wadia Hospitals for Children, Mumbai; PGIMER, Chandigarh; SGRH, New Delhi; Aster CMI Hospitals, Bangalore; Apollo Indraprastha Hospitals, New Delhi) have carried out HSCT for CGD until date. Successful engraftment was documented in 15 patients who received either bone marrow or peripheral blood-derived stem cells. A child who received cord-blood transplantation failed to develop early myeloid engraftment and succumbed to severe infection. This is similar to the experience reported by Morio et al. (58). Four out of six children who received haploidentical transplantation have had a successful engraftment, indicating that haploidentical transplantation is a potential life-saving option in children when completely matched donors are not available. We reiterate that establishment of dedicated pediatric HSCT centres, and government support for patients undergoing HSCT are essential for state-of-the-art management of CGD in developing countries.

To conclude, we document the first multicentric study on clinical and molecular features of 236 patients with CGD from India. To the best of our knowledge, ours is the third largest cohort of patients with CGD documented till date after the multicentric reports from the USA (n = 368) and Europe (n = 429). We have documented a wide spectrum of bacterial and fungal infections and a high incidence of mycobacterial infections in our cohort. Though AR-CGD predominates in our cohort, significant regional differences in molecular spectrum have been observed. Reasons for a high mortality rate (30%) include lack of awareness of CGD among internists and pediatricians, lack of easy access to immunological tests, and paucity of pediatric HSCT services in our country. Establishment of a nation-wide registry for a rare disease like CGD will be needed in future to decipher precise estimate of disease burden in the country and for better identification of barriers in the existing diagnostic and management strategies.

## Data Availability Statement

The data sets presented in this study can be found in online repositories. The names of the repository/repositories and accession number(s) can be found in the article/**Supplementary Material**.

## Ethics Statement

Ethical review and approval was not required for the study on human participants in accordance with the local legislation and institutional requirements. Written informed consent to participate in this study was provided by the participants’ legal guardian/next of kin. Written informed consent was obtained from the individuals for the publication of any potentially identifiable images or data included in this article.

## Author Contributions

PV, MSu, DS, AJi, AnG, SL, PP, AK, PT, AP, VG, MD, SB, CG, HK, DM, MSi, RR, RU, FN, BG, HL, MKa, AS, SSe, TS, AmG, and SSi—clinical management of patients and follow-up and contribution of clinical data. AR, MSh, JS, GK, SC, BS, RM, VA, MKu, GH, UB, PK, MM, AJa, FN, KV, MD, KC, KI, OO, SN, and YL—laboratory work-up of patients and contribution of laboratory data. PV, MSu, MSh, and SL—preparation of first draft and literature review. AR, PV, and SSi—critical review and editing of manuscript. AR and PV—final approval of manuscript. All authors contributed to the article and approved the submitted version.

## Conflict of Interest

The authors declare that the research was conducted in the absence of any commercial or financial relationships that could be construed as a potential conflict of interest.
