# Supplementary material for: Clinical, Immunological, and Molecular Profile of Chronic Granulomatous Disease: A Multi-Centric Study of 236 Patients From India
Source: Front Immunol. 2021 Feb 25;12:625320. doi: 10.3389/fimmu.2021.625320 (PMC7946827; doi:10.3389/fimmu.2021.625320)
Supplement: Supplementary file 1 [file Table_1.docx]

**Supplementary Table 1. Comparison of clinical, and laboratory characteristics between p47^phox^ and p67^phox^ deficiency**

| **Parameter** | **p47^phox^ deficiency (n=55)** | **p67^phox^ deficiency (n=21)** | **p value** |
| --- | --- | --- | --- |
| Median age at onset of infections (IQR)^a^ | 14 months (8- 36) | 5 months (0.1- 26) | 0.056 |
| Median age at diagnosis ^a^ | 32 months (12 - 90) | 22.5 (4.5- 108) | 0.361 |
| Median delay in diagnosis of CGD ^a^ | 10.5 months (1- 34) | 5.5 (1.5- 26.2) | 0.947 |
| Median follow-up ^a^ | 1.2 years (0.1- 8) | 0.75 (0.01 – 2.5) | 0.099 |
| Median stimulation index in DHR ^a^ | 1.61 (1.09- 2.24) | 1.43 (1.07- 2.23) | 0.651 |
| Number of patients with >3 infectious episodes^b^ | 8 | 6 | 0.326 |
| Number of patients with >3 episodes of Pneumonia^b^ | 3 | 0 | 0.545 |
| Number of patients with mortality ^b^ | 7 | 8 | 0.025 |

^a^Mann-Whitney U test

^b^Fisher’s exact test or chi-squared test
