# Supplementary material for: Clinical, Immunological, and Molecular Profile of Chronic Granulomatous Disease: A Multi-Centric Study of 236 Patients From India
Source: Front Immunol. 2021 Feb 25;12:625320. doi: 10.3389/fimmu.2021.625320 (PMC7946827; doi:10.3389/fimmu.2021.625320)
Supplement: Supplementary file 2 [file Table_2.docx]

**Supplementary Table 2. Molecular defects identified in the present cohort of CGD**

| S. No. | Exon/Introns | cDNA/nucleotide change | Protein change | Type of variant | Reference |
| --- | --- | --- | --- | --- | --- |
| *CYBB* | | | | | |
| **1** | Exon 5 | c.433G>A | p.Gly145Arg | Missense | 15 |
| **2** | Exon 5 | c.469C>T | p.Arg157Ter | Nonsense | 15 |
| **3** | Exon 11 | c.1449G>A | p.Trp483Ter | Nonsense | 15 |
| **4** | Exon 3 | c.271C>T | p.Arg91Ter | Nonsense | 15 |
| **5** | Exon 8 | c.868C>T | p.Arg290Ter | Nonsense | 15 |
| **6** | Exon 10 | c.1291G>A | p.Ala431Thr | Missense | 15 |
| **7** | Exon 3 | c.217C>T | p.Arg73Ter | Nonsense | 50 |
| **8** | Intron 6 | c.675−1G>T | del exons 6_7 | Splice site | 30 |
| **9** | Exon 13 | c.1619_1626dupAAGCCTTG | p.Ala543LysfsTer7 | Duplication | Present study |
| **10** | Exon 4 | c.271C>T | p.Arg91Ter | Nonsense | 15 |
| **11** | Promoter | c.-65C>T |  | Promoter | 50 |
| **12** | Exon7 | c.714_715insTA | p.His239TyrfsTer4 | Insertion | Present study |
| **13** | Exon 8 | c.868C>T | p.Arg290Ter | Nonsense | 15 |
| **14** | Exon 3 | c.170C>A | p.Ala57Glu | Missense | 50 |
| **15** | Exon 10 | c.1075G>A | p.Gly359Arg | Missense | 50 |
| **16** | Exon 11 | c.1321_1323delTTC | p.Phe441TyrfsTer130 | Deletion | Present study |
| **17** | Intron 10 | c.1314+1G>A | p.Ile385SerfsTer63? (del exon 10?) | Splice site | 50 |
| **18** | Exon 8 | c.868C>T | p.Arg290Ter | Nonsense | 15 |
| **19** | Exon 6 | c.442C>T | p.Gln148Ter | Nonsense | 50 |
| **20** | Exon 5 | c.426delT | p.Glu143AsnfsTer18 | Deletion | Present study |
| **21** | Exon 3 | c.252G>A | p.Ser48_Ala84del (del. exon 3) | Splice site | 50 |
| **22** | Exon 10 | c.925G>A | p.Glu309Lys | Missense | 50 |
| **23** | Intron 2 | c.141+5_+6delGT | p.Leu16_Gly47del? (del exon 2?) | Splice site | 50 |
| **24** | Exon 9 | c.1049dupT | p.Phe351LeufsTer2 | Duplication | Present study |
| **25** | Intron 9 | c.1152-1G>A | p.Ile385Ter | Splice site | 50 |
| **26** | Exon7 | c.755_756delGA | p.Gly252GlufsTer31 | Deletion | 50 |
| **27** | Exon 12 | c.1546T>C | p.Trp516Arg | Missense | 50 |
| **28** | Intron 6 | c.675−1G>T | del exons 6_7? | Splice site | 30 |
| **29** | Exon 12 | c.1498G>A | p.Asp500Asn | Missense | 50 |
| **30** | Exon 11 | c.1329G>A | p.Trp443Ter | Nonsense | 50 |
| **31** | Exon 1 | c.2T>G | p.Met1Arg | Missense | 50 |
| **32** | Exon 9 | c.960dupA | p.Val321SerfsTer27 | Insertion | Present study |
| **33** | Exon 11 | c.1415delG | p.Gly472AlafsTer30 | Deletion | 50 |
| **34** | Exon 9 | c.1038delT | p.Glu347ArgfsTer39 | Deletion | 50 |
| **35** | Exon 3 | c.252G>A | p.Ser48_Ala84del | Splice site (del. exon 3) | 50 |
| **36** | Exon 3 | c.252G>A | p.Ser48_Ala84del | Splice site (del. exon 3) | 50 |
| **37** | Exon 7 | c.771_777delCCCAATC | p.Pro258LeufsX8 | Deletion | 18 |
| **38** | Exon 11 | c.1418del T | p.Phe473SerfsTer28 | Deletion | 18 |
| **39** | Exon 4 | c. 271C>T | p.Arg91Ter | Nonsense | 15 |
| **40** | Exon 7 | c.676C>T | p.Arg226Ter | Nonsense | 50 |
| **41** | Exon 5 | c.388C>T | p.Arg130Ter | Nonsense | 50 |
| **42** | Exon 3 | c.217C>T | p.Arg73Ter | Nonsense | 50 |
| **43** | Exon 11 | c.1426G>A | p.Trp453Ter | Nonsense | 50 |
| **44** | Exon 5 | c.388C>T | p.Arg130Ter | Nonsense | 50 |
| **45** | Exon 3 | c.252G>A | p.Ser48_Ala84del | Splice site (del. exon 3) | 50 |
| **46** | Exon 5 | c.475A>T | p.Arg159Ter | Nonsense | 18 |
| **47** | Exon 12 | c.1546T>C | p.Trp516Arg | Missense | 50 |
| **48** | Exon 5 | c.475A>T | p.Arg159Ter | Nonsense | 18 |
| **49** | Exon 11 | c.1336_1337insG | p.Asp447GlyfsTer6 | Insertion | Present study |
| **50** | Exon 7 | c.736_737insA | p.Ile248AsnfsTer36 | Insertion | 50 |
| **51** | Exon 10 | c.1154T>G | p.Ile385Arg | Missense | 50 |
| **52** | Exon 5 | c.456T>A | p.Tyr152Ter | Nonsense | 50 |
| **53** | Exon 9 | c.1093del | p.Leu365CysfsTer21 | Deletion | Present study |
| **54** | Exon 3 | c.215_216del | p.Cys72SerfsTer30 | Deletion | Present study |
| **55** | Intron 4 | c.338-5T>A |  | Splice site | Present study |
| **56** | Exon 7 | c.760dupA | p.Ile254AsnfsTer30 | Insertion | 50 |
| **57** | Exon 5 | c.469C>T | p.Arg157Ter | Nonsense | 50 |
| **58** | Exon 7 | c.760dupA | p.Ile254AsnfsTer30 | Insertion | 50 |
| **59** | Exon 3 | c.252G>A | p.Ser48_Ala84del | Splice site (del. exon 3) | 50 |
| **60** | Exon 7 | c.676C>T | p.Arg226Ter | Nonsense | 50 |
| **61** | Exon 7 | c.760dupA | p.Ile254AsnfsTer30 | Insertion | 50 |
| **62** | Exon 5 | c.388C>T | p.Arg130Ter | Nonsense | 50 |
| **63** | Exon10 | c.1234G>A | p.Gly412Arg | Missense | 50 |
| *NCF1* | | | | | |
| **1** | Exon 2 | c.75_76delGT | p.Tyr26HisfsTer26 | Deletion | 51 |
| **2** | Exon 2 | c.75_76delGT | p.Tyr26HisfsTer26 | Deletion | 51 |
| **3** | Exon 2 | c.75_76delGT | p.Tyr26HisfsTer26 | Deletion | 51 |
| **4** | Exon 2 | c.75_76delGT | p.Tyr26HisfsTer26 | Deletion | 51 |
| **5** | Exon 2 | c.75_76delGT | p.Tyr26HisfsTer26 | Deletion | 51 |
| **6** | Exon 2 | c.75_76delGT | p.Tyr26HisfsTer26 | Deletion | 51 |
| **7** | Exon 2 | c.75_76delGT | p.Tyr26HisfsTer26 | Deletion | 51 |
| **8** | Exon 2 | c.75_76delGT | p.Tyr26HisfsTer26 | Deletion | 51 |
| **9** | Exon 2 | c.75_76delGT | p.Tyr26HisfsTer26 | Deletion | 51 |
| **10** | Exon 2 | c.75_76delGT | p.Tyr26HisfsTer26 | Deletion | 51 |
| **11** | Exon 2 | c.75_76delGT | p.Tyr26HisfsTer26 | Deletion | 51 |
| **12** | Exon 2 | c.75_76delGT | p.Tyr26HisfsTer26 | Deletion | 51 |
| **13** | Exon 2 | c.75_76delGT | p.Tyr26HisfsTer26 | Deletion | 51 |
| **14** | Exon 2 | c.75_76delGT | p.Tyr26HisfsTer26 | Deletion | 51 |
| **15** | Exon 2 | c.75_76delGT | p.Tyr26HisfsTer26 | Deletion | 51 |
| **16** | Exon 2 | c.75_76delGT | p.Tyr26HisfsTer26 | Deletion | 51 |
| **17** | Exon 2 | c.75_76delGT | p.Tyr26HisfsTer26 | Deletion | 51 |
| **18** | Exon 2 | c.75_76delGT | p.Tyr26HisfsTer26 | Deletion | 51 |
| **19** | Exon 2 | c.75_76delGT | p.Tyr26HisfsTer26 | Deletion | 51 |
| **20** | Exon 2 | c.75_76delGT | p.Tyr26HisfsTer26 | Deletion | 51 |
| **21** | Exon 7 | c.604C>T | p.Arg202Ter | Nonsense | 52 |
| **22** | Exon 2 | c.75_76delGT | p.Tyr26HisfsTer26 | Deletion | 51 |
| **23** | Exon 2 | c.75_76delGT | p.Tyr26HisfsTer26 | Deletion | 51 |
| **24** | Exon 2 | c.75_76delGT | p.Tyr26HisfsTer26 | Deletion | 51 |
| **25** | Exon 2 | c.75_76delGT | p.Tyr26HisfsTer26 | Deletion | 51 |
| **26** | Exon 2 | c.75_76delGT | p.Tyr26HisfsTer26 | Deletion | 51 |
| **27** | Exon 2 | c.75_76delGT | p.Tyr26HisfsTer26 | Deletion | 51 |
| **28** | Exon 2 | c.75_76delGT | p.Tyr26HisfsTer26 | Deletion | 51 |
| **29** | Exon 2 | c.75_76delGT | p.Tyr26HisfsTer26 | Deletion | 51 |
| **30** | Exon 2 | c.75_76delGT | p.Tyr26HisfsTer26 | Deletion | 51 |
| **31** | Exon 2 | c.75_76delGT | p.Tyr26HisfsTer26 | Deletion | 51 |
| **32** | Exon 2 | c.75_76delGT | p.Tyr26HisfsTer26 | Deletion | 51 |
| **33** | Exon 2 | c.75_76delGT | p.Tyr26HisfsTer26 | Deletion | 51 |
| **34** | Exon 2 | c.124C>T | p. Arg42Trp | Missense | 53 |
| **35** | Exon 2 | c.124C>T | p. Arg42Trp | Missense | 53 |
| **36** | Exon 2 | c.75_76delGT | p.Tyr26HisfsTer26 | Deletion | 51 |
| **37** | Exon 2 | c.75_76delGT | p.Tyr26HisfsTer26 | Deletion | 51 |
| **38** | Exon 2 | c.75_76delGT | p.Tyr26HisfsTer26 | Deletion | 51 |
| **39** | Exon 2 | c.75_76delGT | p.Tyr26HisfsTer26 | Deletion | 51 |
| **40** | Exon 2 | c.75_76delGT | p.Tyr26HisfsTer26 | Deletion | 51 |
| **41** | Intron 6 | c.574+1G>A | (del. exon 6+7?) | Splice site | [Present](http://www.ncbi.nlm.nih.gov/sites/entrez?cmd=Retrieve&db=PubMed&list_uids=22924696&dopt=Abstract) Study |
| **42** | Exon 2 | c.75_76delGT | p.Tyr26HisfsTer26 | Deletion | 51 |
| **43** | Exon 7 | c.581G>A | p.Trp194Ter | Nonsense | 54 |
| **44** | Deletion involving exons 2-10 | | | Deletion | Present study |
| **45** | Exon 2 | c.75_76delGT | p.Tyr26HisfsTer26 | Deletion | 51 |
| *NCF2* | | | | | |
| **1** | Exon 9 | c.835_836delAC | p.Thr279GlyfsTer16 | Deletion | 51 |
| **2** | Intron 13, Exon-12 | c.1179–2A>T, c.1099C>T | p.Gln367Ter | Splice-site, Nonsense (Compound heterozygous) | 51 |
| **3** | Exon 9 | c.835_836delAC | p.Thr279GlyfsTer16 | Deletion | 51 |
| **4** | Exon 9 | c.835_836delAC | p.Thr279GlyfsTer16 | Deletion | 51 |
| **5** | Exon 9 | c.835_836delAC | p.Thr279GlyfsTer16 | Deletion | 51 |
| **6** | Exon 9 | c.835_836delAC | p.Thr279GlyfsTer16 | Deletion | 51 |
| **7** | Intron 5 | c.501+1_+8delGTAAGCGT |  | Splice site | Present study |
| **8** | Exon 13 | c.1148_1149delTG | p.Leu382ArgfsTer8 | Deletion | 18 |
| **9** | Exon 9 | c.835_836delAC | p.Thr279GlyfsTer16 | Deletion | 51 |
| **10** | Exon 2 | c.229C>T | p.Arg77Ter | Nonsense | 51 |
| **11** | Exon 9 | c.835_836delAC | p.Thr279GlyfsTer16 | Deletion | 51 |
| **12** | Exon 9 | c.835_836delAC | p.Thr279GlyfsTer16 | Deletion | 51 |
| **13** | Exon 9 | c.835_836del | p.Thr279GlyfsTer16 | Deletion | 51 |
| **14** | Intron 13 | c.1178+1G>A; c.1167C>A | p.His389Gln | Splice site; Missense (Compound heterozygous) | 55, 56 |
| **15** | Exon 9 | c.834_835delAC | p.Thr279GlyfsTer16 | Deletion | 51 |
| **16** | Exon 12 | c. 1148_1149 TG del | p.Leu382ArgfsTer8 | Deletion | 18 |
| **17** | Exon 2 | c. 73G>A | p.Ala25Thr | Missense | 18 |
| **18** | Exon 5 | c.550C>T | p.Arg184Ter | Nonsense | 51 |
| **19** | Exon 4 | c.419delC | p.Ala140ValfsTer5 | Deletion | Present study |
| **20** | Exon 5 | c.550C>T | p.Arg184Ter | Nonsense | 51 |
| **21** | Exon 3 | c.(257+1_258-1)_(366+1_367-1)del |  | Deletion (exon 3) | Present study |
| *CYBA* | | | | | |
| **1** | Exon 2-4 | c.(58+1_59-1)_(287+1_288-1)del |  | Deletion (exons 2-4) | 51 |
| **2** | Exon 1 | c.27G>A | p.Trp9Ter | Nonsense | 22 |
| **3** | Exon 3 | c.157G>C | p.Glu53Gln | Missense | 18 |
| **4** | Exon 4 | c.269G>A | p.Arg90Gln | Missense | 51 |
| **5** | Exon4 | c.268C>T | p.Arg90Trp | Missense | 57 |
| **6** | Exon 4 | c.269G>A | p.Arg90Gln | Missense | 51 |
| **7** | Exon 2 | c.70G>A | p.Gly24Arg | Missense | 58 |
| **8** | Exon 6 | c.385G>A | p.Glu129Lys | Missense | 51 |
| **9** | Intron 4 | c.288-1G>T | p.Leu97ArgfsTer68 | Splice site (del. exon 5) | 51 |
| **10** | exon 4 | c.268C>T | p.Arg90Trp | Missense | 51 |
| **11** | exon 4 | c.268C>T | p.Arg90Trp | Missense | 51 |
| **12** | Exon 5 | c.269G>A | p.Arg90Gln | Missense | 51 |
