# Supplementary material for: Clinical, Immunological, and Molecular Profile of Chronic Granulomatous Disease: A Multi-Centric Study of 236 Patients From India
Source: Front Immunol. 2021 Feb 25;12:625320. doi: 10.3389/fimmu.2021.625320 (PMC7946827; doi:10.3389/fimmu.2021.625320)
Supplement: Supplementary file 3 [file Table_3.docx]

**Supplementary Table 3. Comparison of our cohort with other multicentric or large patient cohorts of Chronic Granulomatous Disease that are reported previously**

| **Study, Year, Country** | **No. of patients**  **Male/Female** | **Mutations** | **Pattern of infections** | **Non-infective complications** | **HSCT** | **Outcome of HSCT** | **Overall**  **Mortality** |
| --- | --- | --- | --- | --- | --- | --- | --- |
| Winkelstein et al, 2000, USA (1) | Total: 368  Male: n=316 (86%)  Female: n=52 (14%) | *CYBB*: n=259 (70%)  AR: n=81 (22%)  *NCF1*: n = 45 (12%)  *NCF2*: n =10 (3%)  *CYBA*: n =7 (2%)  Insufficient data: n =19 (5%)  Unknown: n =28 (8%) | Pneumonia: 290 (79%)  Sub cutaneous abscess: 156 (42%)  Liver abscess: 98 (27%)  Peri-rectal abscess: 57 (15%)  Brain abscess: 12 (3%)  Suppurative adenitis: 195 (53%)  Osteomyelitis: 90 (25%)  Bacteraemia/fungemia: 65 (18%)  Cellulitis: 18 (5%)  Meningitis: 15 (4%) | 1. Lupus syndromes  DLE: n=10  SLE: n= 2  2. Obstructive lesions  Gastric outlet n=57  Esophageal outlet n=3  Urinary outlet n=37  3. Colitis/enteritis n=64  4. ITP n=5  5. Myasthenia gravis n=1  6.Chorioretinitis n=8 | N.A. | N.A. | Died: n=65 (17.6%)  X- linked: n=54 (21.2  AR: n=7 (2%)  unknown: n=4 (1%) |
| Jones et al, 2008, United Kingdom (35) | Total: 94  Male: n=87(93%)  Female: n=7(7%) | *CYBB:* n=69 (81%)  AR: n=16 (19 %)  Unknown:  n = 9 (9.5%) | Pneumonia: 49  Suppurative adenitis:38  Liver abscess: 27  Septicaemia: 22  Osteomyelitis: 11  Lung abscess: 9 | Colitis: 35 (37%)  Oesophageal stricture (5)  Pyloric stenosis (6)  Gingivitis, stomatitis, oral ulceration: 23 (24%)  Chorio- retinal abnormalities: 9 (39%) | Patients transplanted: n=12 (13%)  One patient had gene therapy | N.A. | N.A. |
| Van den Berg et al, 2009, European cohort  Multi-national cohort (6) | Total :429  Male: n=351 (82%)  Female: n=78 (18%) | *CYBB*:  n=290 (67%)  AR: n=139 (33%)  *NCF1*:  n=69 (49%)  *NCF2*: n=11(8%)  *CYBA*:  n=22 (16%)  Unknown: n=37(27%) | **Site of infection (episodes)**  Lung: 634  Skin and subcutis: 341  Lymph node: 622  Gastro-intestinal: 643  Liver: 240  Kidney/urinary tract: 139  Septicaemia: 111  Ear: 84  Bone: 84  Eye: 84  Joint: 35  Brain 34 | Perianal fissure/ abscess: 21%  Stomatitis, apthae and gingivitis and caries: 11%  Colitis: 9%  Pyloric outlet obstruction: 2%  Autoimmune: 26 (6%)  Discoid lupus: 18 (4%)  Rheumatoid arthritis: 2  Systemic lupus erythematosus, dermatomyositis, sacroiliitis, idiopathic thrombocytopenia and autoimmune hepatitis- all 1 case each | N.A. | N.A. | Died: n=84 (20%) |
| Fattahi et al, 2011, Iran (36) | Total: 93  Male: n= 58 (62%)  Female: n= 35 (38%)  Consanguinity rate: n=72  (77.4%) | *CYBB*: n=12 (13%)  AR:  n= 81 (87%)  *NCF1* n=45 (48%)  *NCF2* n=5 (5%)  *CYBA* n=17 (18%)  Unknown: n=14(15%) | BCGitis: 55.9%  **Site of infections**  Pulmonary: 53 (57%)  Skin: 50 (53.8%)  Gastrointestinal: 31 (33.3%)  Skin and appendages: 50 (53.8%)  Lymphoreticular: 61 (65.6%)  Musculoskeletal: 28 (30.1%)  Hepatobiliary: 27 (29%)  Genitourinary: 14 (15.1%)  CNS: 10 (10.8%) | SLE/Discoid lupus: 5 (16.1%)  (1 XL, 4AR)  Autoimmune enteropathy:1(XL)  Rheumatoid arthritis: 3 AR (3.2%)  ITP: 1 (1%) AR  Chorioretinitis: 1 (1%) AR | N.A. | N.A. | Died: n= 9 (10%)  XL: n=3 (3.2%)  AR: n=6 (6%)  p47^phox^ n=3  p22^phox^ n= 3 |
| Koker et al, 2013, Turkey (34) | Total: 89  Male: n=64  Female: n=25 | CYBB: n=34 (71%)  AR:  n=50 (56%)  *NCF1*:  n= 17 (19%)  *NCF2*:  n=13 (14%)  *CYBA*:  n=20 (22%)  Unknown: n=5 (5%) | BCGitis: 13 (22.5%)  Pneumonia: 90%  Suppurative lymphadenitis: 76%  Liver/ deep seated abscess: 72%  Cutaneous/ subcutaneous infections: 39%  Osteomyelitis: 25%  Sepsis: 31%  UTI: 25%  Enteric infections: 25%  Otitis media: 21%  Perianal abscess: 22 %  Brain abscess: 18% | IBD: 5 (5.6%)  (XL-4, AR-1)  Stomatitis and gingivitis (38)  Reactive arthritis: 3 (33%) AR  FMF symptoms with uveitis: 1 AR  ITP: 1 AR  Pericardial effusion without infective aetiology: 1 XL, 1AR  Autoimmune hepatitis: 2 XL | N.A. | N.A. | Died: n = 9 (10%)  (3 XL, 6 AR)  Mortality percentage-10% |
| Oliveira-Junior et al, 2015, LASID Registry (37) | Total: 71  Males: n=58 (82%)  Females: n=13 (18%) | *CYBB:*  n= 53(75%)  *NCF1:*  n=16 (22.5%)  *CYBA:*  n=2 (3%) | Recurrent pneumonia: 76.8%  Lymphadenopathy: 59.4%  Granulomata: 49.3%  Skin infections: 42%  Chronic diarrhoea: 41.9%  Otitis: 29%  Sepsis: 23.2%  Abscesses: 21.7%  Recurrent urinary tract infections: 20.3%  Osteomyelitis: 15.9% | Chronic inflammatory colitis: 19.4% | No BMT details |  | No details in the article |
| Marcino et al, 2015, USA  (44) | Total: 268 | CYBB:  n= 184 (68%)  *NCF1*:  n= 70 (26%)  *NCF2*:  n= 6 (2%)  *CYBA*:  n= 8 (3%)  *NCF4*:  n= 1 (0.3%) | Lung infection: 87%  Liver abscess: 32%  Lymphadenitis: 25%  Organisms:  *Aspergillus*: n= 125 (46%)  *Burkholderia:* n=46 (17%)  *Serratia*: n= n=42 (15%)  *Nocardia:* n=49 (18%)  Staphylococcus: n=90 (33%) | N.A | N.A | N.A | Overall Mortality  : n = 47 (17%) |
| Wolach et al, 2017, Israel (53) | Total :84  Male: n=67(80%)  Female: n=17(20%)  Consanguinity rate – 64% | *CYBB*:  n=32 (38%)  AR:n=52 (62%)  *NCF 1*:  n=26 (31%)  *NCF 2*: n=16 (19%)  *CYBA:* n=10 (12%) | Pneumonia:74%  Supp.lymphadenitis:61%  Cutaneous/subcutaneous: 51%  Liver abscess: 39%  Gingivo-stomatitis: 27%  Entero-colitis: 25%  Sepsis: 20%  Osteomyelitis: 20%  Perianal abscess:18%  Supp.otitis media: 15%  CNS infections: 5%  Genito-urinary infections: 10%  Disseminated BCGosis: 2% | 1.Granulomas  Total :68%  Liver :39%  Lung: 25%  CNS:5 %  Urinary tract outflow 5%  Intestinal: 5%  Esophageal: 4%  Gastric: 1%  Bladder: 1%  Testicles;1%  2.Crohn’s disease: n=2  3.T cell ALL: n=1  4.Carcinoma of uterine cervix: n=1 | n= 21 (25%)  8- gp91^phox^  AR-13  3- p47^phox^  9-p67^phox^  1- p22^phox^  (16 fully matched sibling donor, 2 fully matched unrelated donor, 2 haploidentical donor) | Post- transplant died: n=16 (19%) | Overall long-term survival- 81% |
| Zhou et al, 2018,  Shanghai, China (32) | Total: 169  Male: n=162 (96%)  Female: n=7 (4%) | *CYBB*: n=150 (89%)  *NCF1*:  n=5 (3%)  *NCF2*:  n=7 (4%)  *CYBA*: n=7(4%) | Lung: 95.9%  Lymph nodes: 58.4%  Skin: 45.4%  GIT: 43.1%  Perianal: 38.5%  Blood stream: 28.5%  UTI: 10%  Soft tissue: 10.8%  Abdominal: 7.7%  Bone: 7.7%  Liver: 4.6%  CNS: 4.6%  Middle ear: 2.3%  Spleen: 0.1% | Ulcer and colitis: 3 (2.3%)  Recurrent oral ulcer: 7 (5.4%)  Anaemia: 72 (55.4%)  Liver function impairment: 45 (34.6%) | HSCT total: 31  21: Cord blood stem cell transplantation | HSCT total: 31  Follow up details available: 28  Died: 6 (21.4%)  4- Died of severe sepsis in post-transplant period  2-Cause unclear | Follow up details available: 131  Overall mortality: 36.6% |
| Gao et al, 2019,  Beijing, China (47) | Total 159 (7.8:1) | *CYBB*: n=132 (83%)  *CYBA*: n=7 (4%)  *NCF1*: n = 4 (2.5%)  *NCF2* : n =4 (2.5%)  No gene mutations detected: n= 6 (4%) | BCGitis: 16 (10%)  Pneumonia: 4 (30%)  Perianal abscess: 9 (6%)  Cutaneous abscess: 5 (3%)  Diarrhoea: 9 (6%)  Omphalitis: 1(1%)  Pustular eruption:12 (8%)  Fever: 92 (59%)  Lymphadenitis: 8(5%) | N.A. | HSCT: n=25 (16%)  (*CYBB*) | Died of GVHD: n=3 (2%)  Died of post-transplant infection: n=1 | Died: n=52 (32%) |
| Wang et al, 2019, China (48) | Total :114  Male: n=109(96%)  Female: n=5(4%) | *CYBB*: n=95(83%)  AR: n=13 (11%)  *NCF1*:  n=3 (3%)  *NCF2*:  n=2 (2%)  *CYBA*: n=8(7%)  Unknown: n=6 (5%) | Recurrent fever: 100%  Pneumonia: 92.1%  Abscess: 73.7%  Lymphadenitis: 58.8%  Diarrhoea: 45.6%  Sepsis: 30.7%  Hepatosplenomegaly:  58.8%  Oral thrush: 16.7%  UTI: 7%  Osteomyelitis: 4.4%  Definitive BCGosis : n=54 (48%) | Inflammatory bowel disease or Crohn’s disease: 11.4% | Patients transplanted: n=29 (25%)  26: successfully transplanted  (14- unrelated donor stem cells, unrelated cord blood- 3, Related donor (sibling)-7, Related donor (father)-4, Related donor (carrier sister)-1 | Died of rejection: n=2 (2%) | Overall mortality: n=6  (24%) |
| Blancas-Galicia et al, 2020, Mexico (41) | Total- 93  Male: n=88 (95%)  Female: n=5 (5%) | *CYBB*: n= 64 (69%)  *NCF1*: n=7  (7.5%)  *NCF2*: n=7  (7.5%)  *CYBA*: n=5 (5.5%) | BCGitis: 47 (53.4%)  BCGosis: 28 (31.8%)  Pneumonia: 74 (79.6%)  Gastroenteritis: 56 (60.2%)  Sepsis: 47 (50%)  Skin and soft tissue infection: 41 (44%)  UTI: 37 (39%)  Pulmonary abscess: 22 (23%)  Sinusitis: 20 (21%)  Otitis media: 20 (21%)  Hepatic abscess: 15 (16%)  Perianal abscess: 15 (16%)  Osteomyelitis: 14 (15%)  Peritonitis: 9 (10%) | HLH: 12 (13%)  Inflammatory granuloma: 52 (56%)  Ulcerative stomatitis/ gingivitis: 25 (27%)  Chronic diarrhea: 9 (9.6%)  Autoimmune cytopenia: 7 (7.5%)  Ocular autoimmunity: 2 (2%)  Kawasaki disease: 1(1%)  SLE: 1 (1%)  Antiphospholipid syndrome: 1(1%)  Autoimmune thyroiditis: 1(1%)  Autoimmune hepatitis: 1 (1%)  Non-Hodgkin lymphoma- 1 | Total: n = 15 (16%)  11 -gp91^phox^  2-p67^phox^  1- p47^phox^  1-mutation  was not identified | Successful engraftment  n = 7 (7.5%)  Graft failure n = 8 (9%)  GVHD: n=4 (4.5%)  4 (4.5%) patients died from serious infections after transplantation | Total died: n= 37 (40%)  X linked: n= 28(30%)  AR: n = 4 (4. 5%) |
| Present study | Total: 236  Male: n=172 (73%)  Female: n=64 (27%) | CYBB: n= 77 (32.6%)  AR: n=97 (41.1%)  *NCF1*:  n=55 (56.7%)  *NCF2*:  n=22 (22.6%) | Mycobacterial infections: 44 (18.6%)  BCGitis: 13 (5.5%)  BCGosis: 4 (1.7%)  Pneumonia: 71.6%  Lymphadenitis: 31.6%  Skin and subcutaneous abscess: 23.7%  Blood-stream infection: 9%  Osteomyelitis: 8.6%  Liver abscess: 7.2%  Lung abscess: 2.9%  Meningoencephalitis: 2.5%  Splenic abscess: 1.7%  Brain abscess: 0.9% | Colitis: 12 (5.1%)  Lung granuloma: 16 (6.8%)  Secondary HLH: 6 (2.5%)  Chilblains: 2  HLA-B-27 arthritis: 1  Kawasaki disease with coronary artery aneurysm: 1  Chronic kidney disease: 1  Intestinal obstruction: 1 | HSCT n=23 (9.7%)  8: Matched related donors    3: Matched unrelated donors  7: Haplo-identical  1: Umbilical cord blood | Complete donor chimerism: 16  6: Died  [2: GVHD  1: Primary graft failure  1: CNS complication] | Outcome details available n=174  Died: 67 (38.5% %)  (X-Linked: 27  AR: 28  Undetermined: 12) |

*Abbreviations:* XL- X-linked; AR- Autosomal recessive; HSCT- Hematopoietic stem cell transplantation; BMT- Bone marrow transplantation; DLE- Discoid lupus erythematosus; SLE- Systemic lupus erythematosus; ITP- Immune thrombocytopenic purpura; IBD- Inflammatory bowel disease; HLH- Hemophagocytic lymphohistiocytosis; GVHD- Graft versus host disease; CNS- Central nervous system
